# Increase of Small-Pox

**Published:** 1854-03

**Authors:** 


*Increase of Small-Pox.*— It is becoming too evident that this exanthem is on the increase in this country. During January there were 132 deaths from it in the city of New York, and during the first week of February it formed the largest item in the bill of mortality, excepting consumption, there being 47 deaths from variola, to 55 from tuberculosis.

It is prevailing, we believe, in every city of the land, and to no inconsiderable extent in the rural districts. It does not, however, manifest that tendency to rapid and wide spread propagation which marked its course before the days of Jenner. Still, the number of deaths by it is so large, that it is evidently only "scotched not killed," a disease still extant, alive and active, seeking every open avenue for spreading, consigning many to the grave, and leaving its mark on the faces of those who survive it.

This increased prevalence of the disease has given rise to the supposition that vaccinia is losing its efficacy in prophylaxis, and is no longer the sure preventive we have been accustomed to consider it.

Undoubtedly too much confidence has been placed in vaccinia by a portion of the profession. Very recently we saw a well-marked vaccine scar upon a patient then thickly covered with the discrete variety of small-pox. But we would not argue from this that this man had not, at some previous time, been thoroughly protected.

It has become very evident, that in order to insure the full benefit of vaccinia, it should be occasionally repeated, the manner of its pustulation forming the criterion of the individual's safety. The people, who rest secure in the safety of a single vaccination, should be taught that it is necessary, at certain intervals, to recur to the means of prevention.

It has been urged that this would be looked upon as a trick upon the part of the physician to add to his income, and that it would detract from the faith of the people in the resources of professional skill. Such arguments should never apply. The physician has no right to consider consequences in such a case. It is his duty to inform his patients of the necessity of re-vaccination, leaving to them the responsibility of neglecting it. And it is further his duty to bear such a reputation as a man of honor, as would raise him above the likelihood of any such trivial imputations as that implied by the opponents of vaccination on politic grounds.

But vaccination is by no means so universal as to lead us to expect from it the entire extinction of small-pox. A very large proportion of the public are never vaccinated, while a still larger portion are still resting in fancied security upon a vaccination performed many years before.

The fatality of small-pox is worthy of note. That 132 cases of it should die, in a single month, in the city of New York, indicates, not only its prevalence, but its malignant character. It is, however, impossible to judge very closely about it, while we know nothing of the circumstances under which it was treated.

Our readers will, perhaps, recollect the brief notice we gave, some months since, of M. J. B. Cayol's attack upon "typhoidism," and the declaration that as small-pox was banished by vaccinia, its place was filled by typhus, by a certain law of compensation in disease. It is a little unfortunate for M. Cayol, that typhoid diseases are really on the increase in this country; and, on the other hand, it is no less unfortunate for the law of compensation, that typhoid and variola are both prevalent synchronously, and increasing *pari passu*. H.

